# Sampling strategies for sugarcane using either clonal replicates or diverse genotypes can bias the conclusions of RNA-Seq studies

**DOI:** 10.1590/1678-4685-GMB-2022-0286

**Published:** 2023-04-03

**Authors:** Victor Hugo Mello, Ana Letycia Basso Garcia, Fernando Henrique Correr, Guilherme Kenichi Hosaka, Monalisa Sampaio Carneiro, Gabriel Rodrigues Alves Margarido

**Affiliations:** 1 Universidade de São Paulo, Escola Superior de Agricultura Luiz de Queiroz, Departamento de Genética, Piracicaba, SP, Brazil.; 2 Universidade Federal de São Carlos, Centro de Ciências Agrárias, Departamento de Biotecnologia e Produção Vegetal e Animal, Araras, SP, Brazil.

**Keywords:** Differential gene expression, vegetatively propagated crops, biological replicates, sugarcane transcriptomics, missing samples

## Abstract

A key procedure for ensuring statistical confidence in differential gene expression analyses is to use biological replicates to compare distinct groups. Biological replicates allow the estimation of the residual variation in the gene expression levels among samples of a given experimental condition. In sugarcane, it is possible to obtain an estimate of residual variability at two levels: among samples of distinct genotypes of the same experimental treatment, or clonal replicates of the same genotype. The sequencing costs are often a limitation to leveraging both these levels in the same study, stressing the relevance of efforts to determine an appropriate experimental design. We aim to investigate this question by comparing the transcriptional profiles of young sugarcane culms with different sucrose levels using both sampling strategies. Our results show that clonal replicates provided enough statistical power to identify nearly three times more deferentially expressed genes than the more diverse strategy. However, it resulted in potentially less meaningful biological results, because many of the significant genes were likely related to the particular genotype of choice, rather than representing a common expression profile for the compared groups. This study supports the development of sound experimental designs in new studies regarding differential expression for sugarcane.

## Introduction

The genus *Saccharum* comprises six species, of which *S. spontaneum* and *S. robustum* are the only wild representatives, spread over a large area in Asia and Indonesia, and the others are domesticated species - *S. oﬀicinarum*, *S. barberi*, *S. sinense*, and *S. edule*. The first sugarcane hybrids were obtained from the crossing of *S. oﬀicinarum* and *S. spontaneum*, followed by backcrossing to *S. oﬀicinarum*, such that they inherited the high sugar yield from the former species and the pathogen resistance, adaptability, and increased vigor of the latter ( Irvine, 1999; Piperidis et al., 2010). Sugarcane cultivation accounts for 86% of the worldwide production of sugar, despite the increasing allocation of its juice for ethanol production. Moreover, the sugarcane residue after juice extraction, called bagasse, is a byproduct that can be used for energy generation and production of bioplastics ( Aguilar et al., 2019; OECD/FAO, 2020). The crop is a renewable source of fuel and presents a significant advantage over fossil fuels due to the reduced emission of greenhouse gases ( Goldemberg, 2008).

Sugarcane breeding programs usually rely on a few recurrent crosses between elite parents or wild germplasm to produce genotypes with desired traits, mainly sugar or fiber yield and resistance to abiotic and biotic stresses ( Heinz and Tew, 1987; de Souza Barbosa et al., 2002; Jackson, 2005). Therefore, each breeding program develops new hybrid varieties per cycle, a few of which are commercially released ( Cursi et al., 2022). From the milieu of available genotypes, scientific investigations in sugarcane are often based on a few elite lines that are recurrently used. For instance, two genome assemblies were recently published for the hybrids R570 - a major model in sugarcane genomic studies - and SP80-3280 ( Garsmeur et al., 2018; Souza *et al.,* 2019). These efforts are remarkably relevant for sugarcane genomic research, given the complexity of its genome.

Also, these hybrids show a large variation in chromosome number and genome constitution. *S. oﬀicinarum* (2n = 8x = 80) and *S. spontaneum* (2n = 40-128), the parental species, have high levels of ploidy and complex genomes per se ( Bremer, 1925; Panje and Babu, 1960). Chromosome number multiplicity and molecular evidence have led to the acceptance of the basic number of x = 8 for *S. spontaneum* ( Liu et al., 2016); however, the description of a wild accession with x = 10 brought a new panorama to the evolutionary history of the genus ( Meng et al., 2020). These facts reveal an intricate set of hurdles concerning the understanding of sugarcane genomics, which must be considered for data-driven experiments. Hence, many sugarcane studies focus on transcriptomic data to avoid the challenges imposed by its genome (at least partially).

More specifically, the use of phenotypic trait variation between genotypes is a common approach found in differential expression studies. Gene expression studies in sugarcane have been conducted using a single genotype to represent the phenotypic group ( Casu et al., 2007; Papini-Terzi et al., 2009; Casu *et al.,* 2015; Vicentini et al., 2015; Dharshini et al., 2016; Rody et al., 2019; Nawae et al., 2020; Selvi et al., 2020), as well as multiple genotypes per group ( Papini-Terzi *et al.,* 2009; Ferreira et al., 2016; Thirugnanasambandam et al., 2017; Hoang et al., 2017; Correr et al., 2020). Biological replicates provide more accurate estimates of transcript abundances when comparing samples from two treatment levels. Clones from the same genotype are subject to variability in their expression levels due to factors such as interactions with the environment and other organisms. Still, the transcriptional variation within clones is expected to be smaller when compared to plants from different genotypes, which decreases the dispersion of gene quantification estimates. Statistical parameters such as means of expression levels and their residual variances are the main variables considered in modern differential expression tests, which highlights the relevance of the choice of approach for performing these studies. While the use of clones renders a more homogeneous set of samples, and consequently more statistical power to detect differences in expression between groups, it also restrains the set of samples to a limited number of genotypes. Nevertheless, other concerns about the comparison of contrasting groups in RNA-Seq analysis were raised, such as minimum sample sizes and the use of technical replicates for ensuring reproducibility ( Conesa et al., 2016).

Here, we evaluate the influence of using clonal replicates or multiple genotypes in the contrasting groups when performing differential gene expression analysis. The comparison of approaches we propose relies both on quantitative estimates of differentially expressed genes and qualitative functional enrichment tests. We aim to present an information-based criterion for selecting biological replicates for further experiments using RNA-Seq, particularly for sugarcane, whose genomic properties can deviate dramatically among genotypes.

## Material and Methods

### Biological material and RNA-Seq

The genotypes chosen for this study are part of the Brazilian Panel of Sugarcane Genotypes, located in Araras - Brazil. They were selected from 254 genotypes to represent elite lines and commercial hybrids used in Brazil, as well as ancestral species of the *Saccharum* complex ( Medeiros et al., 2020). First, for the strategy based on diverse genotypes (SBDG), we selected 12 genotypes and separated them into four groups with three members each. This categorization divided genotypes based on their content of soluble solids, measured in ºBrix: VLB (Very Low ºBrix), LB (Low ºBrix), HB (High ºBrix), VHB (Very High ºBrix). The phenotypic characterization of this panel of genotypes, including the content of soluble solids, is described in B arreto et al. (2019). Next, for the strategy based on clones (SBC), we chose one representative of each group and used three clonal replicates of these genotypes to represent the corresponding phenotypic groups ( Table 1).

**Table 1 -:** Genotypes selected to compose each ºBrix group for the strategy based on clones (SBC) and based on diverse genotypes (SBDG). In the former strategy, we sampled the immature internode +1 of three clonal replicates (R1, R2, and R3) for each genotype per group, and samples from three different genotypes per group for the latter. The genotypes IN84-58, F36-819, R570, and SP80-3280 were represented in both strategies, using samples from different plants.

Group	Genotype	# of samples in SBC	# of samples in SBDG	Soluble solids (ºBrix ± s.d.)
VLB	Krakatau	0	1	12.03 ± 2.02
	SES205A	0	1	13.99 ± 3.14
	IN84-58	3	1	14.78 ± 1.89
LB	Criolla Rayada	0	1	16.53 ± 1.37
	IJ76-317	0	1	17.68 ± 1.75
	F36-819	3	1	18.05 ± 1.51
HB	White Transparent	0	1	19.60 ± 2.01
	RB92579	0	1	19.77 ± 1.29
	R570	3	1	20.69 ± 1.24
VHB	White Mauritius	0	1	21.25 ± 1.38
	SP80-3280	3	1	21.29 ± 1.88
	RB835486	0	1	21.85 ± 1.86

Immature culms (internode +1) from all 24 plants were collected in June 2016, in Araras, followed by extraction of total RNA with the RNeasy Plant Mini Kit (Qiagen) according to the manufacturer's recommendations. We prepared the RNA-Seq libraries of polyadenylated transcripts using the TruSeq Stranded mRNA LT (Illumina) protocol. These libraries were sequenced in a HiSeq 2500 equipment (Illumina), resulting in paired-end reads 2x100 bp long. The 12 libraries of the SBC were sequenced in three lanes, in combination with other samples not used in this study, with final sequencing depth corresponding to eight samples per lane. For the SBDG we used a single lane exclusively for the twelve samples.

### Downsampling and quality control

Because the SBC data showed higher average counts per sample, we first carried out a downsampling step. This procedure aimed to balance the differences in sequencing depth between both datasets, achieving the same amount of information for the two strategies. For that, we applied the sample function of the Seqtk suite (https://github.com/lh3/seqtk), using as parameters a fixed random seed -s100 and the probability of removing a read proportional to the ratio of the average read counts of SBDG and SBC samples. After that, we used the programs Cutadapt v1.18 ( Martin, 2011) and Trimmomatic v0.38 ( Bolger et al., 2014) to: i) trim residual sequences of Illumina adapters from raw reads; ii) remove base pairs with Phred score less than 20 in a window of 5bp; iii) trim the first 13bp of each read; and iv) remove paired reads shorter than 50 bp ( Table S1).

### *De novo* transcriptome assembly and functional annotation

We chose to perform a *de novo* transcriptome assembly based on all samples to minimize the potential effect of representation biases on genes and alleles from different genotypes. For that, we used the libraries after downsampling and quality control as input to Trinity v2.8.0 ( Grabherr et al., 2011), using the default parameters except for the normalization by readset. Functional annotation was carried out with blastx and blastp ( Altschul et al., 1990) significant hits (e-value < 10^−5^) against the Swiss-Prot database, using ORFs identified in the transcriptome with Transdecoder (https://github.com/TransDecoder/TransDecoder). We also annotated protein domains using hmmscan v3.2.1 ( Eddy, 2009) with the Pfam database. All these sources of information were compiled with the software Trinotate v3.1.1 (https://github.com/Trinotate/Trinotate) to produce the final annotation. This reference was further assessed by the identification of conserved orthologs among green plants and monocotyledons, using the software BUSCO v3 ( Simão et al., 2015) with databases in OrthoDB10.

Next, we used the quasi-mapping strategy of salmon v0.12.0 ( Patro et al., 2017) to quantify the expression of the assembled transcripts, separately for each sample. The transcriptome file was used to build an index with a k-mer size of 31 bp, with the additional parameters of GC bias correction and validate mappings to achieve higher mapping rates and confidence levels. We then summarized transcript counts per gene and normalized to obtain expression estimates in counts per million (CPM). CPM values were used to quantify gene expression for all downstream analyses.

### Comparison of differential expression results with the full dataset

For differential expression analyses, we initially excluded lowly expressed genes, by filtering out genes that did not show a CPM greater than one for at least three samples. We did this filtering individually for each strategy, resulting in different sets of filtered genes for SBC and SBDG. Next, the following steps were repeated with the same criteria for both strategies, using the edgeR package ( Robinson et al., 2010). We normalized the gene counts with the trimmed mean of M-values method ( Robinson and Oshlack *,* 2010) and built MDS (Multidimensional scaling) plots using the top 2,000 genes with the greatest pairwise variation between samples. 

For statistical tests of differential expression, we considered a model for gene counts parametrized as follows,

$$Y_{g,i}\;\sim \;NB\left(\mu_{g,i},\;\Phi_{g}\right)\;$$ (1)

for sample *i* in an experimental group, gene *g*, π_g,i_ the fraction of gene counts per gene and sample, dispersion *Φ*_*g*_, libraries size *N*_*i*_, average counts *µ*_*g,i*_ = N_*i*_ π_*g,i*_ , and variance *Φ*_*g*_ = π_*g,i*_ ( *1+π*_*g,i*_ Φ_*g*_ ). The common dispersion is the squared Biological Coeﬀicient of Variation (BCV), which considers the common dispersion from all genes. The use of a local regression of genewise dispersion provides an additional level of information for dispersion estimates for each gene. As a result, *Φ*_*g*_ represents a compromise between the dispersion of counts for gene *g* and the borrowed genewise dispersion from genes with close average CPM.

We designed three orthogonal contrasts to test for differential expression for each gene, namely VLB × VHB.HB.LB, corresponding to the null hypothesis $H_{0}:\pi_{g,VLB}=\frac{\pi_{g,VHB}+\pi_{g,HB}+\pi_{g,LB}}{3}$ VHB × HB.LB to H0 :πg,VHB = $\frac{\pi g,HB\;+\;\pi g,LB}{2}\;\;$ and HB × LB to $H_{0}\pi_{gHB}=\pi_{g,LB}\;$ . A likelihood ratio test was performed for each combination of gene, contrast, and strategy to identify the differentially expressed genes (DEGs), with p-values adjusted by the false discovery rate (FDR, Benjamini and Hochberg, 1995) at a 0.05 significance threshold.

Using the sets of DEGs and the annotated transcriptome, we performed functional enrichment analyses considering the frequency of Gene Ontology (GO) terms in the background reference and each set. Because the average gene length may vary among GO categories, care was taken to calculate effective gene lengths, based on the average length of genes in each sample weighted by their expression levels. We used the goseq package ( Young et al., 2010) to perform the functional enrichment test for each represented GO term (p < 0.01, after adjusting for multiple tests with the FDR approach).

**Impact of missing samples on differential expression results**

In addition to using all samples of each strategy, we also analyzed the effect of systematically removing samples on the differential expression results. This procedure can provide a better understanding of the effect of individual samples on the downstream analysis, as well as establishing a comparison between this approach and the use of full data. We have developed a method to compare different combinations of subsets of samples, under the condition that valid combinations must have at least two samples per group. This restriction is necessary because minimal replication per group is required to properly calculate gene dispersions, even if the estimates are less accurate. Because there are four groups with three samples each, 255 combinations exist, all of which were individually tested for differential expression with the same contrasts previously designed. The number of combinations of different numbers of removed samples is given by the binomial factor:

$$n_{i}=\;\left(\begin{array}{c} g\\ i \end{array}\right)k^{i}$$ (2)

in which *k* represents the number of samples per group ( *k = 3*), *g* represents the number of groups ( *g = 4*), and *i* represents the number of removed samples, ranging from one to four. For each combination, we removed genes with low expression levels (CPM > 1 in less than two samples) and recorded the differential expression result as one of the following categories: *(a)* upregulated, *(b)* downregulated, *(c)* not significant, or *(d)* filtered out. One result was obtained for each gene, combination of samples, contrast and sampling strategy. We applied the same workflow for performing differential expression and functional enrichment tests as in the full data analyses.

Among all tested combinations of samples in our subsampling evaluation, one of special interest is that composed of the eight genotypes present exclusively in SBDG. The strategy based on clones comprised a single genotype per group of soluble solids content, namely, IN84-58, F36-819, R570, and SP80-3280 ( Table 1). For SBDG, we chose another eight genotypes in addition to these, which we call exclusive genotypes of SBDG, specifically SES205A, Krakatau, Criolla Rayada, IJ76-317, White Transparent, RB92579, White Mauritius, and RB835486. We also performed analyses of differential expression with this subset of samples.

### Code and data availability

All the scripts are available at the Github repository (github.com/victor-h14/BiologicalReplicates). The raw RNA-Seq reads are available at the European Nucleotide Archive, with all the samples from the BioProjects PRJEB44302 for SBDG and PRJEB40481 for SBC.

## Results

### Gene identification in the sugarcane transcriptome

The objective of our study was to compare the sampling strategies based on clones (SBC) and based on diverse genotypes (SBDG) for RNA-Seq studies. Because of that, we performed a *de novo* transcriptome assembly using all 24 samples from both sampling strategies to use as a reference for gene quantification. The resulting transcriptome included 598,874 transcripts for a total of 262,281 assembled genes ( Table S2). Reads from both strategies were evenly represented in the assembly, with an average mapping rate of 76.5% among samples ( Table S1). Genes had an average size of 932.63 bp and the transcript N50 was 1,687 bp. The majority of genes had a single corresponding transcript isoform (64.3%).To assess the quality of our transcriptome, we checked the representation of conserved single-copy orthologs from Viridiplantae and Liliopsida clades - green plants and monocotyledons, respectively. We identified 95.1% of the 430 orthologs conserved in green plants without sequence fragmentation. For the set of orthologs in monocots, 93.1% of 3,278 orthologs were fully represented.

### Comparison of differential expression results between strategies

Our goal was to compare both datasets based on the results from the differential expression and functional enrichment analyses. We followed a standard procedure for these tasks using edgeR and goseq. After library sizes were normalized, the quantification outputs still contained a large amount of lowly expressed genes. We selected genes with CPM > 1 in at least three samples, for each strategy separately, resulting in different sets of kept genes for SBC and SBDG. The former presented 42,566 genes after filtering, and 41,934 remained in the latter ( Figure S1).

**Figure 1 -:**
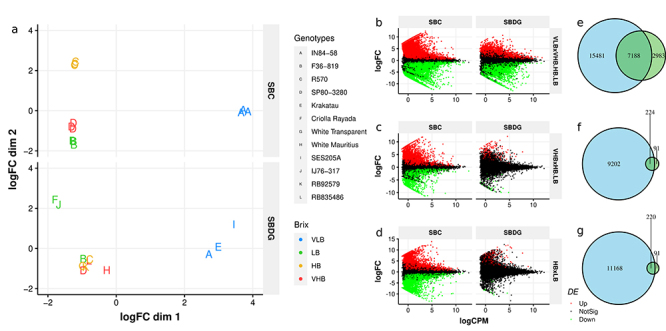
Gene expression patterns and differentially expressed genes for both sampling strategies. ( **a**) Multidimensional scaling (MDS) plot showing pairwise distances between samples based on the most divergent genes for each pair. The panels represent the MDS plot for the strategy based on clones (SBC) and the strategy based on diverse genotypes (SBDG), respectively. ( **b**, **c**, **d**) Mean-difference plot grid showing differentially expressed genes for all contrasts and strategies. Fold changes (logFC) and average expression levels in counts per million (logCPM) are shown in base 2 log scales. The rows indicate the three orthogonal contrasts, while the columns correspond to the strategies of sampling biological replicates. Colors represent the result of differential expression tests (p < 0.05, after FDR correction for multiple tests). ( **e**, **f**, **g**) Differentially expressed genes shared by the strategies based on clones and on diverse genotypes. The diagrams represent the number of genes detected as significantly differentially expressed in the contrasts. The strategy based on clones is in blue, while the strategy based on diverse genotypes is in green. The figures represent orthogonal contrasts VLB × HB.LB.VHB ( **b**, **e**), VHB × HB.LB ( **c**, **f**), and HB × LB ( **d**, **g**).

An initial exploratory investigation allowed for assessing the main characteristics of expression profiles with a multidimensional scaling plot ( Figure 1A). For the SBC, we observed clustering of replicates from each genotype, indicating high similarity in the expression profiles of clonal replicates. As expected, the first dimension of the plot separated replicates of genotype IN84-58 from the remaining groups, reflecting their contrasting genetic backgrounds. On the other hand, the biological variance of gene expression was much higher in the diverse approach than in the clone approach. In SBDG we found a little overlap of samples from the same phenotypic group, except for the VLB genotypes, which again were isolated from the others by differences in the first component. No clear pattern was observed for genotypes of VHB and HB. In fact, only two LB *S. oﬀicinarum* accessions, Criolla Rayada and IJ76-316, clustered apart.

The MDS analysis provided a broad view of the overall patterns of transcription abundances for the set of samples, but did not allow a closer assessment of individual genes. We then used the differential expression testing approach for a detailed investigation of the transcriptome expression profiles. We arranged the four groups of samples into three orthogonal contrasts. Hence, we conducted three tests of differential expression for each gene. The quantity of DEGs identified via the SBC largely surpassed that of SBDG for all contrasts, especially in VHB × HB.LB and HB × LB ( Figure 1B, File S1). In these two contrasts, we can observe a mass of significant DEGs in relatively low absolute logFC values for SBC. Conversely, only a few DEGs were significant for SBDG, even for genes showing fold changes of large magnitude. We identified non-significant genes even at |logF C| > 10, standing for more than a thousand-fold variation of read counts. The non-significance of genes with high values of logFC is possible because the adopted likelihood model for gene abundance considers gene counts and variance within groups for the likelihood ratio test. We can thus (at least partly) attribute the lower number of DEGs for the SBDG to the higher residual variance in gene counts observed with this strategy. An indicator of dispersion with a meaningful interpretation is the Biological Coeﬀicient of Variation (BCV), calculated as the square root of the negative binomial dispersion of counts. The average BCV for all filtered genes of the SBC was 0.087, and 0.440 for the SBDG, representing a five-fold variation between strategies. In addition, for the set of genes retained after filtering for both strategies (37,535 genes), 98% of them showed higher BCV in the SBDG. These numbers reinforce the role of dispersion as a key parameter that distinguishes the approaches regarding differential expression.

The intersection of sets of DEGs between strategies revealed that the majority of genes identified as significant in the SBDG was also significant in the SBC, but the opposite was not true ( Figure 1E-G). About 71% of DEGs detected with the SBDG were shared with the other strategy, for each of the three contrasts. This fact suggests that using more diverse genotypes favored the identification of genes with similar expression patterns among the group members. The observation regarding the high residual variance for VHB x HB.LB and HB x LB also strengthens this hypothesis, because only the more homogeneously expressed genes achieved significance. On the other hand, the use of clones was also able to identify many other genes as differentially expressed, which are possibly genotype-specific and may not be directly associated with the phenotype of interest.

**Figure 2 -:**
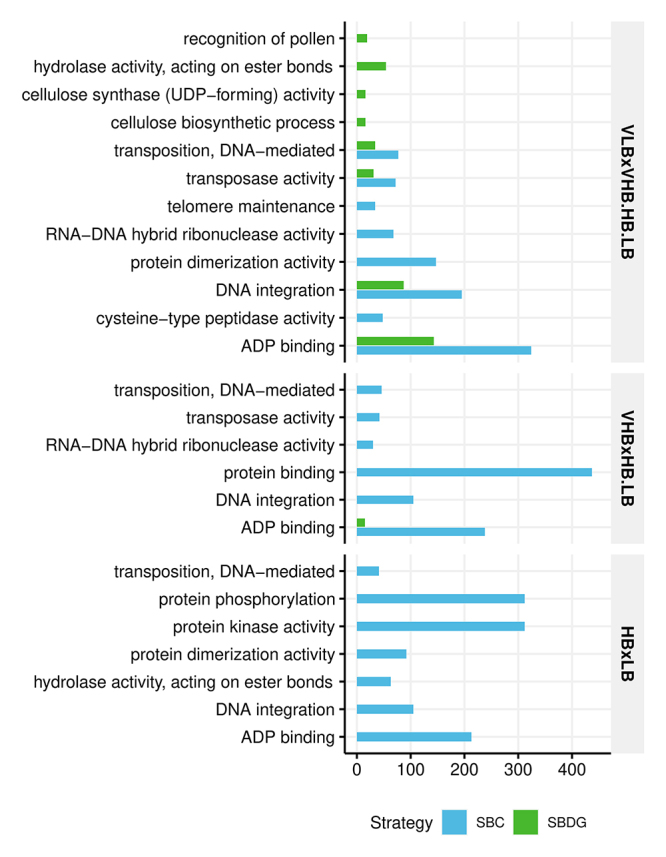
Enriched gene ontology terms by strategy and contrast. There was no significant test result for HB × LB in the strategy based on diverse genotypes (p < 0.01, after FDR adjustment). The numeric axis represents the number of differentially expressed genes for each particular gene ontology term.

The current work presents a systematic analysis of the effects of competing strategies of biological replication over gene expression studies. Our goal is not to provide a biological interpretation of expression patterns, but to justify with biological reasoning the use of each methodology. Therefore, we chose the functional enrichment analysis as a meaningful approach for understanding the consequences of data-mining over the sets of filtered and differentially expressed genes. Within each set of genes that passed the expression filter, we found 12,364 and 11,979 genes containing at least one attributed GO term for SBC and SBDG, respectively ( File S2). Using these genes as a background reference, we performed a functional enrichment analysis to identify GO terms more frequent among DEGs than expected by chance alone ( Figure 2, File S3). For the SBDG, the contrast VHB x HB.LB resulted in only one enriched term (adenosine diphosphate binding), and HB x LB had no enriched GO.

### Assessment of strategies using subsets of samples

When removing a fraction of samples from the experimental design, the average values of gene counts and variance are modified and less precise, such that the resulting set of DEGs may be different. For this reason, we adopted the strategy of systematically removing samples as a validating procedure of the expression results. Also, this approach evaluates the effect of variation on the number of samples per group, such as in unbalanced experiments, or missing samples. Handling mistakes, low volumes of biological material, diﬀiculties in preparing the sequencing libraries, and other unexpected events often cause (random) loss of samples. Because each strategy includes 12 samples divided into four groups, and there must be at least two per group for estimating the dispersion parameter, we could jointly remove a maximum of four samples. These restrictions produced 255 combinations, which were individually tested for differential expression.

**Figure 3-:**
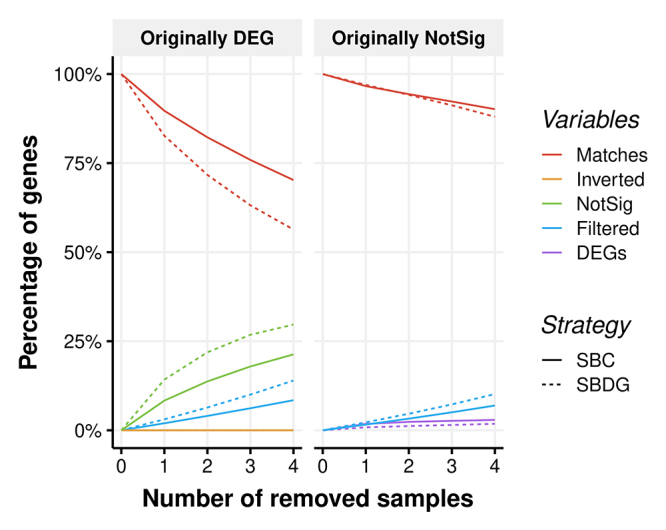
Effect of sample removal on the results of differential expression. The values represented by the continuous (SBC) and dashed (SBDG) curves are the averages of differential expression tests for all possible combinations and contrasts, as a function of the number of removed samples. The red curve indicates the concordant genes in the original and subsampled datasets; in yellow, the differentially expressed genes with inverted results, whether up or downregulated; in green, genes that were not significant due to subsampling; in blue, filtered genes after subsampling; and in purple, genes that appeared as spuriously differentially expressed with subsampling.

We observed that, as the number of removed samples changed from one to four, the more differential expression disagreed with the results obtained with the full dataset. Albeit at low rates, we could identify genes with an inverted result of differential expression, *i.e.*, miscalls of up or downregulation, which occurred from 10^−6^ to 10^−5^ % of genes for the SBC, and from 10^−5^ to 10^−4^ % for the SBDG. Using the original data results as a gold standard (full set of samples), the strategy based on clones showed a relatively lower percentage of false negatives and a higher percentage of false positives - green and purple curves in ( Figure 3), respectively.

**Figure 4 -:**
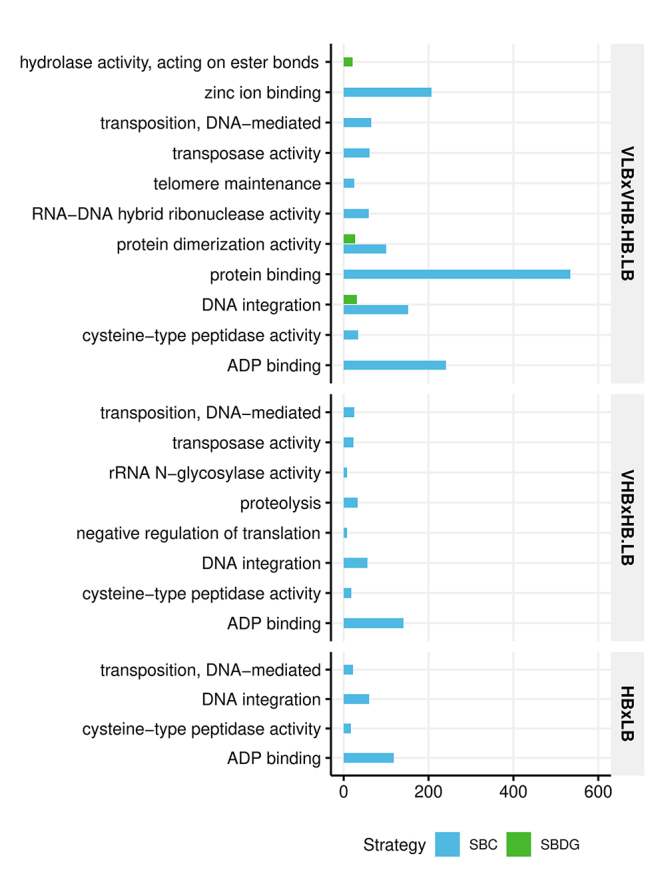
Enriched GO terms by strategy and contrast for the high confidence set of DEGs. This set contains genes with a significant test for differential expression in more than 95% of combinations of samples (p < 0.01, after FDR adjustment).

Because our systematic removal of samples provided a large number of differential expression tests for each gene, we could establish a high confidence set of DEGs - those with at least 95% of tests with the same results ( Table 2). We then used this high confidence set for performing a functional enrichment analysis ( Figure 4, File S4). The enriched GO terms for the full set of DEGs and the high confidence set were essentially different. There were only three enriched terms for the SBDG, of which two had also been detected with the full dataset, and the other was only significant for the SBC. Given the low number of annotated and differentially expressed genes for the contrasts VHB × HB.LB and HB × LB, it was not possible to detect any enriched term for the SBDG. Analyzing exclusively the SBC, nearly 73% of the terms were also enriched in the full dataset for VLB x VHB.HB.LB, 50% for VHB x HB.LB and 75% for HB x LB. Also, the number of enriched terms was high, even with fewer DEGs for the test. Some terms were exclusive for the high confidence set, such as zinc ion binding, proteolysis, and negative regulation of translation. The opposite also occurred, such as for kinase activity.

**Table 2 -:** The differentially expressed genes (DEGs) in the high confidence set. We identified these genes as differentially expressed in at least 95% of the subsampling combinations when removing from one to four samples in each strategy. We considered only the combinations which presented a minimum of two samples per experimental group. For each strategy, we show the total number of DEGs and those annotated with gene ontology terms.

Contrast	SBC DEGs		SBDG DEGs	
	Total	Annotated	Total	Annotated
VLB × VHB.HB.LB	14240	2688	2960	458
VHB × HB.LB	5774	829	44	7
HB × LB	5371	994	34	7

### Contribution of SBDG exclusive genotypes for differential expression

**Figure 5 -:**
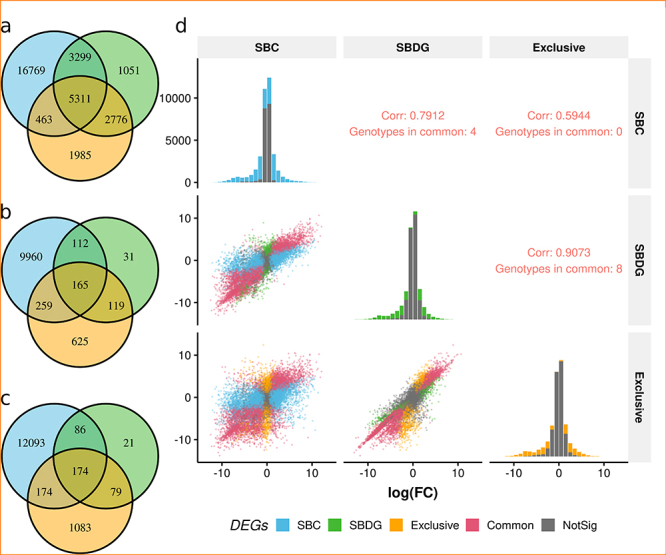
Differentially expressed genes shared by three sets of samples: SBC, SBDG, and SBDG-exclusive genotypes. The method for filtering genes with low expression was the same for the three sets, adopting a more permissive criterion due to the lower number of samples in the exclusive set (CPM > 1 for at least two samples). We only considered the genes passing the low expression filtering criterion in all sets (38,420 genes). The diagrams represent contrasts VLB × HB.LB.VHB ( **a**), VHB × HB.LB ( **b**), and HB × LB ( **c**). Correlation of log fold changes among the two strategies and the set of SBDG-exclusive samples, for contrast VLB × VHB.HB.LB ( **d**). In the main diagonal, the histograms show how the distribution of differentially expressed genes (DEGs) and non-DEGs, as a function of the log fold change (logFC). The classification of genes considered the result of differential expression for all pairwise groups of strategies (SBC, SBDG, and SBDG-exclusive genotypes). Pink points represent genes with a significant test for a given pair of groups, and gray points represent non-significant genes for the same pair.

We compared the DEGs identified in the subgroup of genotypes absent in SBC with the data from SBC and SBDG, using the same parameters for the analysis ( Figure 5). It was possible to observe distinct patterns between the contrast VLB x VHB.HB.LB and the others, regarding the number of DEGs called by each approach. In the first contrast, the total number for SBDG was greater than for the exclusive set, as opposed to the results for the last two contrasts. We also highlight that the larger fraction of DEGs detected in SBDG concentrated in the intersection with the other approaches.

## Discussion

Analyzing the patterns found in the MDS plots, we can infer that transcriptional profiles agreed only partially with the phenotypic assortment of genotypes into four categories of soluble solids content ( Figure 1). This plot also shows a recurrent observation in the other analyses regarding the sharp disparities found between VLB and the other groups. This fact is evident in the separation of samples in Figure 1, the increased number of DEGs from the VLB x VHB.HB.LB tests, when compared to the other contrasts, and the functional enrichment from SBDG ( Figure 2). A likely explanation is the genetic background of the genotypes, because VLB comprises *S. spontaneum* accessions, while VHB, HB, and LB comprise *S. oﬀicinarum* and commercial hybrids. Despite having a genomic contribution from both parental species, commercial hybrids underwent backcrossing to *S. oﬀicinarum* to enhance sugar yield, which makes them more alike to this species in terms of expression. Our result matches the clustering pattern found in the MDS plot for sugarcane leaf samples, in which *S. spontaneum* genotypes separate from the remaining ( Correr et al., 2020). This interpretation also agrees with cytogenetic information from R570, because about 80% of its chromosomes presented similarity to *S. oﬀicinarum* and 10% to *S. spontaneum* ( D’Hont et al., 1996; Garsmeur et al., 2018). Another reasonable explanation for the observed disparity of VLB is the imbalance in chromosome numbers, which remains to be assessed by karyotyping. Changes in ploidy levels and aneuploidy can lead to systematic differences in phenotype and gene expression ( Liqin et al., 2019; Johnson et al., 2020).

This assumption was reinforced by the SBDG contrasts, where VHB × HB.LB and HB × LB showed only a few DEGs. When considering the fraction of significant DEGs in common between strategies using the full dataset, the amount of shared significant tests was nearly constant over the three contrasts in SBDG ( Figure 1). The same was not true for SBC, which had a rate of shared DEGs ranging from 2 to 32%. A feasible explanation is that sugarcane genotypes have high variability of expression among each other, and the use of clones provides enough statistical power to detect it. However, a substantial proportion of these genes might not be actually related to the biological phenomenon of interest, because the lower variability in the SBC led to the identification of DEGs with lower fold-change magnitudes. Extrapolating these results, we can suggest that the SBC was not fully representative of the groups of interest, because of the low agreement of DEGs identified in common with the SBDG.

Several of the identified enriched GO terms fit in molecular mechanisms with no explicit relationship to the accumulation of sugars or carbon partitioning. For instance, the contrast HB × LB in SBC, which represents a direct comparison of the genotypes R570 and F36-819, showed a significant enrichment of kinase activity, which may indeed represent an important mechanism that distinguishes the phenotypes of these plants. However, phosphorylation is a broad molecular mechanism of signal transduction, and it could be related to other processes other than sugar accumulation. Besides, the expression patterns of genes associated with protein phosphorylation were not consistent among the other genotypes. On the other hand, some of the terms found in the SBDG functional enrichment are coherent with observable phenotypic traits, *e.g*., recognition of pollen and cellulose biosynthetic process. Pollen recognition is a potentially vital activity for genotypes in the VLB group, because it is composed uniquely of wild accessions, which probably are prone to perform sexual reproduction without human assistance. Also, the discrepant levels of fiber in VLB × VHB.HB.LB groups corroborate the enrichment of cellulose biosynthetic activity. With the outcomes of functional enrichment for the high confidence set, we could recognize several GO terms in disagreement with the DEGs based on the full set. The terms discussed above such as pollen recognition, cellulose biosynthetic process, and kinase activity were not significant for these high confidence genes. These examples highlight the lack of similar expression patterns among all samples. Besides the biological and residual sources of variation in gene expression quantitation, stochastic processes also contribute to the variance of RNA-Seq data, such as the random sampling of transcripts in library preparation. For SBDG, we could also consider that the genotypes in each group have different contributions to the differential expression result. More precisely, combining a diverse set of genotypes into an experimental group increases the overall variability of expression levels for most genes and modifies the average counts per group. 

We presented a selection of four genotypes for the SBC, which is one particular choice among 81 (3^4^) possible combinations if maintaining the same categories from the SBDG. Examining the wide distribution of genotypes in the MDS plot for SBDG ( Figure 1), we can presume that the choice of genotypes can lead to sharply discordant sets of DEGs. This is a result of the faulty coherence of genotypes inside the groups VHB, HB, and LB. Furthermore, the combination-sensitive set of identified DEGs could drive mistaken conclusions regarding the biological issue of interest. For example, a specific gene might be called differentially expressed due exclusively to the choice of sampled genotypes, instead of representing a general phenomenon for other genotypes with similar phenotypic characteristics. The outcomes of the analyses using subsets of samples reinforce this hypothesis ( Figure 3). We can observe an increasing number of genes with contradictory results of differential expression tests when compared to the full-data tests. This fact implies that simply including or not some genotypes may lead to changes in the list of DEGs. Another result that supports the caveats on genotype choice is the number of DEGs for VHB x HB.LB and HB × LB contrasts ( Figure 5). In the former contrast, the exclusive genotypes of the SBDG showed 625 DEGs that could only be found with these samples, versus 31 in SBDG. The difference was even more prominent for HB × LB. Notably, Criolla Rayada and IJ76-317 are *S. oﬀicinarum* accessions that integrate the LB group, both with a discrepant expression profile according to the MDS analysis. The simple inclusion of F36-819 in this group might have been enough to disrupt the homogeneity detected between the other two genotypes. These observations show how the lack of uniformity in the SBDG genotypes leads to a low number of significant DEGs. Moreover, they indicate that this uniformity may be sensible to the choice of genotypes to form the experimental groups.

The behavior of (mis)matches in the detection of DEGs ( Figure S2, S3) can be helpful to illustrate some properties of each strategy. First, they illustrate the more robust response of the SBC regarding the removal of samples by the lower rate of mismatches. This fact reinforces that SBC showed increased statistical power to detect DEGs. Second, these results also suggest that individual samples can have a determinant role on the identification of differential expression for a considerable number of genes, mainly for the SBDG. As shown in Figure S3, the *n*_*i*_ × *n*_*i*_ grids did not reveal a uniform or linear distribution pattern of the power to detect differential expression. There were both rows and columns densely occupied by DEGs, in patterns contingent on the number of removed samples. They occurred in multiples of 25% for three samples and 33% for four, which correspond to the fractions of combinations without a specific sample. Figure S2 revealed a similar pattern, noticeable by the steep inclines of cumulative distributions for particular mismatch rates.

We suggested that the SBDG yielded fewer DEGs due to combining genotypes with more variable expression patterns than the SBC. Also, our interpretation of the results presented evidence towards the prevalence of more biologically meaningful DEGs for SBDG, instead of simply revealing genotype-specific profiles. However, a feasible criticism over these hypotheses is that using a collection of genotypes per phenotypic group could still lead to genotype-specific DEGs, but for more than one genotype at once. A necessary step to avoid this issue is to choose a diverse set of genotypes for the experimental groups, which should be unrelated and representative of the population of interest. For tackling this question, we performed the complete analysis procedure using the genotypes exclusive to the SBDG, such that we could assess the direct contribution of the genotypes shared with the SBC, the genotypes absent in SBC, and the intersection between them. Interestingly, this analysis showed that the intersection between SBC and the exclusive set concentrated most of the SBDG genes in all contrasts (from 37 to 48% of DEGs). This result agrees with the expectation of a shared set of genes among all 12 genotypes. Moreover, the correlations of logFC in VLB x VHB.HB.LB among the approaches revealed that SBDG had an intermediate pattern for differential expression between SBC and the exclusive set of samples ( Figure 5). Another important observation is that the increased number of samples for SBDG compared to the exclusive set of samples led to a larger number of DEGs in the first contrast and a smaller number in the other two. Thus, we can hypothesize that as the number of genotypes per group increased, the issue of detecting genotype-specific DEGs and genes with reduced biological meaning decreased.

Our results emphasize that sampling strategies are sources of bias in differential expression analysis. This conclusion draws special attention to vegetatively propagated species, as is the case of sugarcane, because many researchers opt to use clones as biological replicates. We suggest that the choice of replication strategy should be planned carefully. This recommendation joins previous guidelines for differential gene expression studies, such as the number of biological samples, library size, and sequencing design ( Conesa et al., 2016; Lamarre et al., 2018).

With the increasing application of next-generation sequencing to investigate complex transcriptomes, such as that of sugarcane, recent studies aim to apply these techniques to unravel the molecular mechanisms controlling several phenotypic traits. However, a single biological replicate in each contrasting group is not enough for performing this sort of analysis, leaving to the researcher the choice of a suitable experimental design. Our study intended to illustrate the strengths and caveats inherent to two sampling strategies for biological replication, namely by using a diverse group of genotypes with common phenotypic characteristics or clones from the same genotype, chosen to be representative of this group. The results provided evidence of discrepancies in (i) quantitative terms, regarding the number of genes detected as differentially expressed, (ii) consistency, when subjected to self-validation using subsampling, and (iii) inferred biological conclusions from the functional annotation of differentially expressed genes. These analyses suggest that the use of clones as biological replicates may yield somewhat restricted results, biased by the particular choice of genotypes. Regardless of these concerns, the direct comparison of two genotypes can still be useful in particular situations. For instance, when there is no need to understand how a broad phenomenon occurs for a species, or when the aim is actually to uncover genotype-specific mechanisms. On the other hand, the presence of a representative set of genotypes within the same group can lead to more reasonable biological conclusions. In any case, it is possible to combine these strategies to refine the level of details, if economically viable. This research offers support for experimental design planning of new studies using differential expression as a method of investigation in sugarcane and other plants with high genomic complexity.

## Supplementary material

The following online material is available for this article:

Table S1 - Processing of sequencing data.

Table S2 - Summary statistics of the *de novo* transcriptome assembly and its annotation with Gene Ontology terms.

Figure S1 - Number of genes kept after filtering out lowly expressed genes.

Figure S2 - Cumulative distribution of identified mismatches.

Figure S3 - Correspondence between differentially expressed genes found by SBC and SBDG per contrast and number of removed samples.

File S1 - Differential gene expression results.

File S2 - List of annotated genes in the transcriptome.

File S3 - Gene Ontology enrichment results.

File S4 - Gene Ontology enrichment for the high confidence set.
