# Supplementary material for: Sampling strategies for sugarcane using either clonal replicates or diverse genotypes can bias the conclusions of RNA-Seq studies
Source: Genet Mol Biol. 2023 Apr 3;46(1):e20220286. doi: 10.1590/1678-4685-GMB-2022-0286 (PMC10075064; doi:10.1590/1678-4685-GMB-2022-0286)
Supplement: Table S1 - [file 1415-4757-GMB-46-1-e20220286-s1.pdf]

Genetics and Molecular Biology
https://doi.org/10.1590/1678-4685-GMB-2022-0286

Supplementary Material to "Sampling strategies for sugarcane using either clonal replicates or diverse genotypes can bias the conclusions of RNA-seq studies"

Table S1 - Processing of sequencing data. Raw data represents the number of reads after downsampling of the SBC dataset. Quality control consisted of trimming and removing low quality paired reads. Mapping rates indicate alignment of reads after quality control against the *de novo* assembled transcriptome. SBC - Strategy based on clones; SBDG - Strategy based on diverse genotypes.

Sample	Raw reads	Reads after quality control	Mapping rate to transcriptome
		**SBC**	
SP80-3280 R1	43,966,734	40,114,252	75.12%
SP80-3290 R2	37,891,704	34,496,514	75.46%
SP80-3280 R3	41,403,168	37,820,702	75.03%
R570 R1	41,005,116	37,893,862	84.22%
R570 R2	44,286,020	40,690,986	75.20%
R570 R3	40,290,944	37,096,532	79.19%
F36-819 R1	43,363,828	39,809,118	76.32%
F36-819 R2	46,032,304	42,445,412	76.61%
F36-819 R3	42,181,506	38,925,260	78.64%
IN84-58 R1	48,151,512	44,359,660	75.04%
IN84-58 R2	48,957,312	45,338,270	79.26%
IN84-58 R3	42,272,278	38,980,420	81.70%
Mean	42,818,054	39,394,770	76.47%
		**SBDG**	
SP80-3280	50,481,854	46,337,774	75.12%
White Mauritius	41,749,608	38,642,584	75.86%
RB835486	38,829,088	35,247,810	77.62%
R570	45,443,304	42,257,284	84.27%
RB92579	45,191,582	41,952,538	76.33%
White Transparent	37,865,194	35,088,926	76.15%

Sample	Raw reads	Reads after quality control	Mapping rate to transcriptome
F36-819	45,598,224	42,274,354	76.62%
Criolla Rayada	40,096,612	40,093,304	76.30%
IJ76-317	40,048,306	37,184,558	76.71%
IN84-58	43,422,964	40,264,200	81.70%
Krakatau	40,349,586	37,372,702	76.92%
SES205A	43,589,964	40,383,156	76.54%
Mean	43,323,910	40,178,752	76.58%
