# Supplementary material for: Sampling strategies for sugarcane using either clonal replicates or diverse genotypes can bias the conclusions of RNA-Seq studies
Source: Genet Mol Biol. 2023 Apr 3;46(1):e20220286. doi: 10.1590/1678-4685-GMB-2022-0286 (PMC10075064; doi:10.1590/1678-4685-GMB-2022-0286)
Supplement: Table S2 - [file 1415-4757-GMB-46-1-e20220286-s2.pdf]

Genetics and Molecular Biology
https://doi.org/10.1590/1678-4685-GMB-2022-0286

Supplementary Material to "Sampling strategies for sugarcane using either clonal replicates or diverse genotypes can bias the conclusions of RNA-seq studies"

Table S2 - Summary statistics of the *de novo* transcriptome assembly and its annotation with Gene Ontology terms. The terms here expressed as *gene* and *transcript* refer to the output of Trinity. Therefore, *gene* comprehends a set of transcripts clustered together in a common de Bruijn graph, sharing sequence identity.

Assembly statistics	Transcriptome	Genes kept in SBC	Genes kept in SBDG
Number of genes	262,281	42,566	41,934
Number of transcripts	598,874	262,099	261,547
Genes with 1 transcript	64.3%	13.5%	12.8%
Genes with 2 transcripts	13.7%	11.8%	11.4%
Genes with 3 transcripts	5.9%	9.3%	9.3%
Genes with 4+ transcripts	16.1%	65.4%	66.5%
Mean transcript length	932.63 nt	1573.62 nt	1567.19 nt
Median transcript length	495 nt	1239 nt	1230 nt
Contig N10	4,295 nt	-	-
Contig N20	3,277 nt	-	-
Contig N30	2,618 nt	-	-
Contig N40	2,124 nt	-	-
Contig N50	1,687 nt	-	-
Total assembled bases	558,526,632 nt	-	-
Number of identified GO terms	1,187	1,169	1,164
Number of annotated genes in the transcriptome	18,667	12,364	11,979
