# Supplementary material for: Sampling strategies for sugarcane using either clonal replicates or diverse genotypes can bias the conclusions of RNA-Seq studies
Source: Genet Mol Biol. 2023 Apr 3;46(1):e20220286. doi: 10.1590/1678-4685-GMB-2022-0286 (PMC10075064; doi:10.1590/1678-4685-GMB-2022-0286)
Supplement: Figure S1 - [file 1415-4757-GMB-46-1-e20220286-s3.pdf]

Genetics and Molecular Biology
https://doi.org/10.1590/1678-4685-GMB-2022-0286

Supplementary Material to "Sampling strategies for sugarcane using either clonal replicates or diverse genotypes can bias the conclusions of RNA-seq studies"

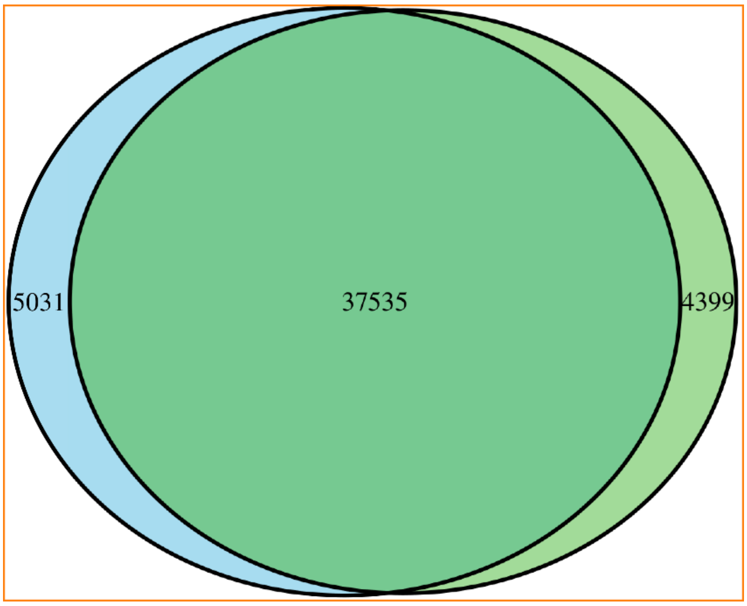

Figure S1 - Number of genes kept after filtering out lowly expressed genes. The strategy based on clones (blue) had 42,566 genes analyzed for differential gene expression, while the strategy based on diverse genotypes (green) had 41,934. 11.8% of SBC and 10.5% of SBDG gene datasets were exclusive.
