# Supplementary material for: Sampling strategies for sugarcane using either clonal replicates or diverse genotypes can bias the conclusions of RNA-Seq studies
Source: Genet Mol Biol. 2023 Apr 3;46(1):e20220286. doi: 10.1590/1678-4685-GMB-2022-0286 (PMC10075064; doi:10.1590/1678-4685-GMB-2022-0286)
Supplement: Figure S2 - [file 1415-4757-GMB-46-1-e20220286-s4.pdf]

Genetics and Molecular Biology
https://doi.org/10.1590/1678-4685-GMB-2022-0286

# Supplementary Material to "Sampling strategies for sugarcane using either clonal replicates or diverse genotypes can bias the conclusions of RNA-seq studies"

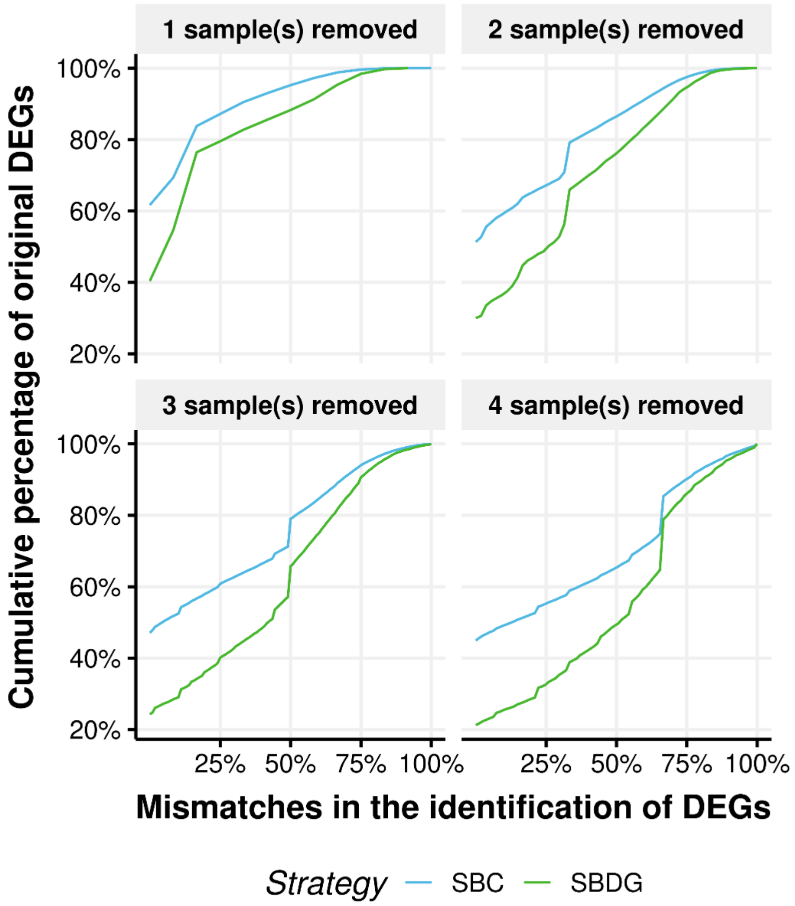


**Figure S2 -** Cumulative distribution of identified mismatches. The total number of combinations of subsets of samples are: $n_1$ = 12, $n_2$ = 54, $n_3$ = 108, and $n_4$ = 81, in which the index $i$ represents the number of removed samples. Thus, the original DEGs and the subsamples may match from 0 to $n_i$ times. We consider a match when a given iteration has the same differential expression result for a gene. Hence, the curves indicate the percentage of DEGs which showed the minimum mismatch rate in the x-axis. For example, with a single sample removed for the SBDG (top left panel), 40% of genes did not have a single mismatch for all twelve combinations, and 80% of genes presented a maximum mismatch rate of 25%.
