# Supplementary material for: Sampling strategies for sugarcane using either clonal replicates or diverse genotypes can bias the conclusions of RNA-Seq studies
Source: Genet Mol Biol. 2023 Apr 3;46(1):e20220286. doi: 10.1590/1678-4685-GMB-2022-0286 (PMC10075064; doi:10.1590/1678-4685-GMB-2022-0286)
Supplement: Figure S3 - [file 1415-4757-GMB-46-1-e20220286-s5.pdf]

Genetics and Molecular Biology
https://doi.org/10.1590/1678-4685-GMB-2022-0286

Supplementary Material to "Sampling strategies for sugarcane using either clonal replicates or diverse genotypes can bias the conclusions of RNA-seq studies"

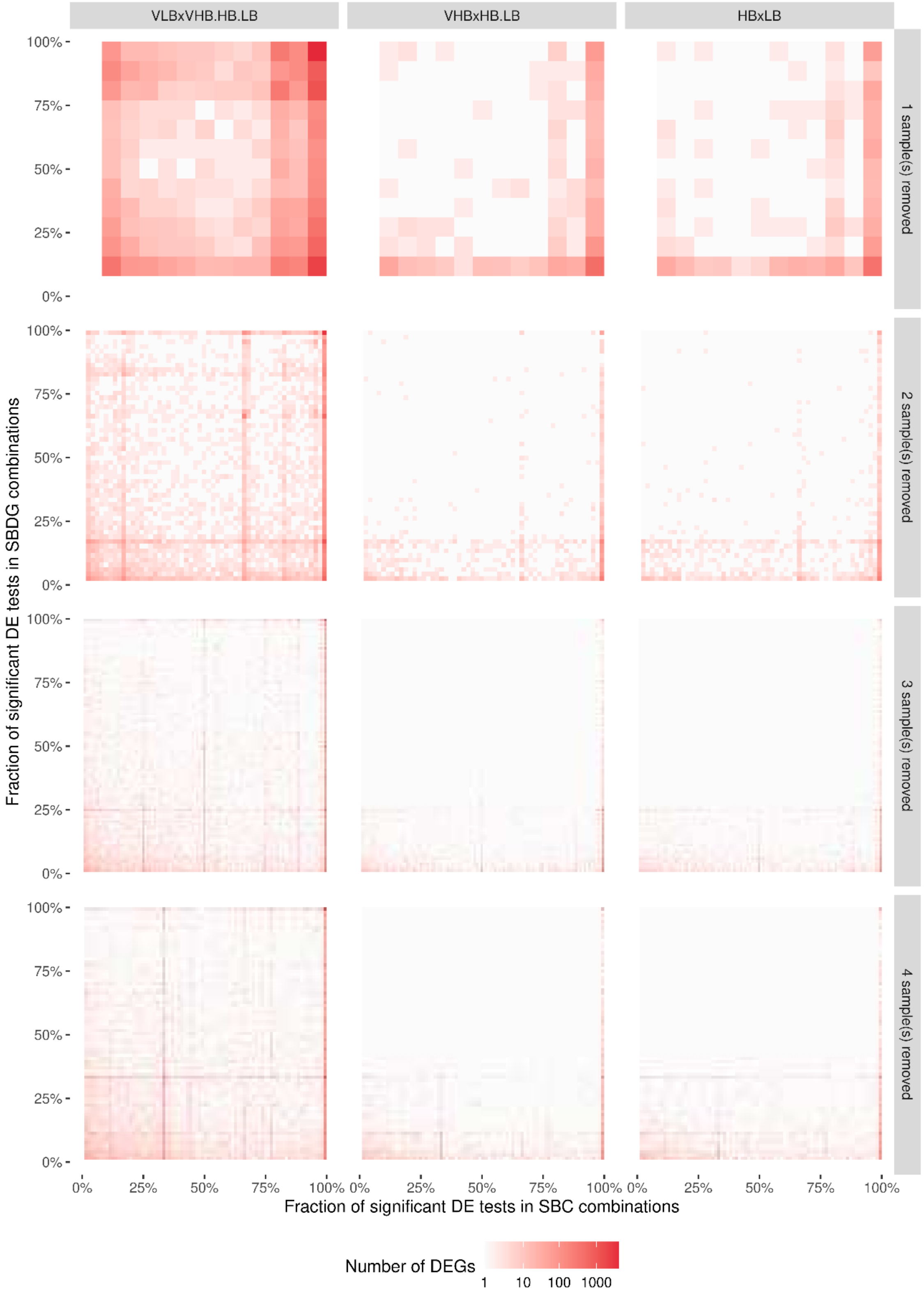

Figure S3 - Correspondence between differentially expressed genes found by SBC and SBDG per contrast and number of removed samples. The number of combinations for i removed sample(s) is n_i, such that each gene ranges from 0 to n_i chances to be called as differentially expressed. The heatmap shows the number of DEGs identified by both strategies, for at least one combination of samples, normalized by n_i.
