# Supplementary material for: Sampling strategies for sugarcane using either clonal replicates or diverse genotypes can bias the conclusions of RNA-Seq studies
Source: Genet Mol Biol. 2023 Apr 3;46(1):e20220286. doi: 10.1590/1678-4685-GMB-2022-0286 (PMC10075064; doi:10.1590/1678-4685-GMB-2022-0286)
Supplement: File S1 - [file 1415-4757-GMB-46-1-e20220286-s6.zip › 1415-4757-GMB-46-1-e20220286-s6/gmb-2022-0286_20230209_suppl6.pdf]

Genetics and Molecular Biology
https://doi.org/10.1590/1678-4685-GMB-2022-0286

Supplementary Material to "Sampling strategies for sugarcane using either clonal replicates or diverse genotypes can bias the conclusions of RNA-seq studies"

The table contains the outcome of the likelihood ratio test after p-value correction for all contrasts in both strategies. The columns logCPM and logFC contain the average expression levels among all the samples included in the contrast and the fold-change of the comparison, respectively, both in the log2 scale. DE summarizes the final result assigned by the test as not significant, upregulated, or downregulated (NotSig, Up, or Down).
