# Supplementary material for: Sampling strategies for sugarcane using either clonal replicates or diverse genotypes can bias the conclusions of RNA-Seq studies
Source: Genet Mol Biol. 2023 Apr 3;46(1):e20220286. doi: 10.1590/1678-4685-GMB-2022-0286 (PMC10075064; doi:10.1590/1678-4685-GMB-2022-0286)
Supplement: File S2 - [file 1415-4757-GMB-46-1-e20220286-s7.zip › 1415-4757-GMB-46-1-e20220286-s7/gmb-2022-0286_20230209_suppl7.pdf]

Genetics and Molecular Biology
https://doi.org/10.1590/1678-4685-GMB-2022-0286

Supplementary Material to "Sampling strategies for sugarcane using either clonal replicates or diverse genotypes can bias the conclusions of RNA-seq studies"

The table shows Gene Ontology terms with a brief description of their function and the list of assembled genes associated with them. The column Ontology groups each term by the three main categories molecular function, biological process, or cell compartment.
