# Supplementary material for: Sampling strategies for sugarcane using either clonal replicates or diverse genotypes can bias the conclusions of RNA-Seq studies
Source: Genet Mol Biol. 2023 Apr 3;46(1):e20220286. doi: 10.1590/1678-4685-GMB-2022-0286 (PMC10075064; doi:10.1590/1678-4685-GMB-2022-0286)
Supplement: File S3 - [file 1415-4757-GMB-46-1-e20220286-s8.zip › 1415-4757-GMB-46-1-e20220286-s8/gmb-2022-0286_20230209_suppl8.pdf]

Genetics and Molecular Biology
https://doi.org/10.1590/1678-4685-GMB-2022-0286

Supplementary Material to "Sampling strategies for sugarcane using either clonal replicates or diverse genotypes can bias the conclusions of RNA-seq studies"

The test evaluates whether a GO category is overrepresented in the set of differentially expressed genes against the full transcriptome annotation. The p-values were corrected using the false discovery rate (FDR) method. numDEInCat and numInCat represent the number of genes for each category in the set of differentially expressed genes and the whole transcriptome, respectively.
