# Supplementary material for: Sampling strategies for sugarcane using either clonal replicates or diverse genotypes can bias the conclusions of RNA-Seq studies
Source: Genet Mol Biol. 2023 Apr 3;46(1):e20220286. doi: 10.1590/1678-4685-GMB-2022-0286 (PMC10075064; doi:10.1590/1678-4685-GMB-2022-0286)
Supplement: File S4 - [file 1415-4757-GMB-46-1-e20220286-s9.zip › 1415-4757-GMB-46-1-e20220286-s9/gmb-2022-0286_20230209_suppl9.pdf]

Genetics and Molecular Biology
https://doi.org/10.1590/1678-4685-GMB-2022-0286

Supplementary Material to "Sampling strategies for sugarcane using either clonal replicates or diverse genotypes can bias the conclusions of RNA-seq studies"

We selected a group of genes with the same result for the differential expression test in more than 95% of the sample removal iterations. GO categories changed not only in the number of genes but also in proportion when compared to the original data. The structure of the file matches the description given for File S3.
